# Peroxisome proliferator-activated receptors (PPARα, PPARγ and PPARβ/δ) gene expression profile on ram spermatozoa and their relation to the sperm motility

**Published:** 2016-03-15

**Authors:** Ali Kadivar, Heidar Heidari Khoei, Hossein Hassanpour, Hamid Ghanaei, Arefeh Golestanfar, Hossein Mehraban, Najmeh Davoodian, Roohollah Dehghani Tafti

**Affiliations:** 1 *Department of Clinical Science, Faculty of Veterinary Medicine, Shahrekord University, Shahrekord, Iran; *; 2 *Research Institute of Animal Embryo Technology, Shahrekord University, Shahrekord, Iran;*; 3 *Students' Research Committee, Department of Biology and Anatomical Sciences, School of Medicine, Shahid Beheshti University of Medical Sciences, Tehran, Iran; *; 4 *Department of Animal Science, Faculty of Agriculture, Shahrekord University, Shahrekord, Iran.*

**Keywords:** Gene expression, Peroxisome proliferator-activated receptors, Ram, Sperm motility

## Abstract

Peroxisome proliferator-activated receptors (PPARs) are a member of nuclear receptors superfamily, which mainly regulate the expression of target genes involved in lipid and energy metabolism. These receptors are divided to three isotypes: PPARα, PPARγ and PPARβ/δ. Each isotype has a distinct tissue distribution relating to the distinct functions. In this study, the mRNA abundance for PPARα, PPARγ and PPARβ/δ was evaluated and compared with high and low motile ram spermatozoa. Semen samples from 6 adult rams were fractionated on a two layer discontinuous Percoll gradient to high and low motile sperm and quantitative parameters of sperm motility were determined by CASA. Total RNA was extracted and the mRNA abundance for each gene was measured by relative quantification technique with Real time PCR. The levels of three isotypes of PPAR transcripts were significantly higher in high motile semen samples using quantitative RT-PCR. Some of sperm motility indices were also significantly correlated with PPARα and PPARγ relative expression. This study revealed the novel association of PPAR gene isotypes with sperm motility. Data from our study suggested PPARs are one of the possible factors that can be studied in male infertility.

## Introduction

Peroxisome proliferator-activated receptors (PPARs) are a member of nuclear hormone superfamily, which mainly regulate the expression of target genes involved in lipid and energy metabolism.^1^ These receptors are divided to three isotypes: PPARα, PPARγ and PPARβ (also known as PPARδ). These receptors are activated by binding of natural ligands, such as polyunsaturated fatty acids and prostaglandin metabolites or by synthetic ligands, such as molecules of the glitazone family.^2^ Each isotype is a product of a separate gene, and each one has a distinct tissue distribution relating to the distinct functions. Numerous functions have been attributed to these receptors. For example PPARγ has been shown to regulate fat mass and cell proliferation,^3^ enhances insulin sensitivity^4^ and modulates inflammatory reactions. In general the PPARs play key roles in the metabolic syndrome and overall health of organisms including lipid metabolism, regeneration of tissues, differentiation, and immune response.^5^

Three PPAR isoforms are suggested to express in several reproductive tissues: gonads (ovary, testis), mammary and pituitary gland, uterus and prostate.^6^^,^^7^ Both somatic and germ cells of the testis express all three PPAR isoforms. The PPARγ and PPARα are widely expressed in interstitial Leydig cells, Sertoli and germ cells.^8^^,^^9^ The action of PPARs in the testis is not completely clear. The expression of PPARα is up regulated by follicle stimulating hormone (FSH),^9^ a key hormone that stimulates protein synthesis, mobilization of energy sources and production of testicular fluid components. PPARα may also regulate the fatty acid composition of phospholipids in germ cells.^7^ The lipid composition of spermatozoa is known to modulate their mobility and its viability.^10^ A study by Aquila *et al*. demonstrated that human sperm express PPARγ and the functionality of this receptor was also investigated.^11^ Up to now many studies have shown that different nuclear receptors, such as progesterone receptor,^12^ androgen and estrogen receptors,^13^^,^^14^ are present in ejaculated human spermatozoa, regulating some cellular processes. It is specified in recent years, the sperm cell expresses various receptor types,^14^ and it also produces their ligands. It suggests a probable role for an autocrine short loop to modulate sperm cell functions independently by the systemic regulation.^15^ Sperm cell need to have a finely regulation of metabolism to affect the changes in signaling pathways encountered during their life, nevertheless there are few findings about the mechanisms underlying the signaling events associated with the change in sperm energy metabolism.

In the present study we show that ram spermatozoa express PPARα, PPARγ and PPARβ/δ, and the amount of expression is associated with sperm motility. 

## Materials and Methods

**Semen samples and spermatozoa preparations. **Testicles from six adult Lori-Bakhtiari rams (1 to 3 years old) were collected from abattoir and transferred to the laboratory at room temperature. Semen collection was carried out in the first 2 hr after the slaughter. Epididymis-testicle complexes were dissected into two parts: testicle, epididymis. Sperm was obtained by slicing the tissue of the cauda epididymis with a scalpel; the fluid was collected by sampler and its volume was estimated. To limit contamination, epididymis samples were carefully dissected free of blood clots and extraneous tissues. Care was taken not to cut blood vessels.

Semen samples were washed with Hepes-buffered tissue culture medium (Hepes TCM; Gibco Life Technologies, Carlsbad, USA) + 10% bovine serum albumin (BSA; Gibco) and sperm suspensions were centrifuged at 500 *g* for 2 min and the supernatant was discarded. This procedure repeated two times. 

**Sperm separation procedures. **Sperm suspension were layered on a two-layer discontinuous Percoll gradient, consisting of 1 mL 45 % (v/v) and 2 mL 90% (v/v) Percoll (Pharmacia Biotech, Uppsala, Sweden) in a 15 mL conical plastic tube (Falcon 2095, Fisher Scientific, Pittsburg, USA). The spermatozoa and gradient were centrifuged at 700 *g* for 20 min. After centrifugation, the separated fractions in the tube were carefully collected in a new set of the tubes, and the volume of each fraction was determined.

**Spermatozoa evaluation. **The assessment of motility parameters was carried out using CASA (Hooshmand Fanavar, Tehran, Iran). Samples were diluted (10 to 20 ×10^6 ^cells per mL) in the same H-TCM medium with 320 mOsm kg^-1^, and kept warm on a 37 ˚C incubator during examination. Then, a 5 µL drop was placed into a Makler counting cell chamber (20 μm depth; Irvine Scientific, Santa Ana, USA) and evaluated. 

The CASA settings were as follows: number of vision fields that were selected, six vision-fields per sample; magnifying power of microscope (object lens), 4×; sperm velocity that can be analyzed, 0-180 μm sec^-1^; image collection speed, 20 frames per sec; analysis time per frame, less than 15 sec. The sperm motility was divided to rapid (class A), slow or sluggish (class B), non-progressive motility (class C), and immotility (class D), all in percentages. The followed sperm motion parameters were studied: curvilinear velocity (VCL), which is the average velocity measured over the actual point to-point track followed by the cell in micrometers per second; straight line velocity (VSL), which represents the average velocity measured in a straight line from the beginning to the end of one track in micrometers per second; average path velocity (VAP), which corresponds to the average velocity of the smoothed cell’s pathway in micrometers per sec; beat cross frequency (BCF) is the frequency at which the sperm cell’s head crosses the sperm cell’s average pathway in Hertz; amplitude of lateral head displacement in micrometers (ALH); the linearity (LIN) which estimates linearity of a curvilinear path in percentage; the wobble (WOB), which is the measure of oscillation of the actual path about the average path; straightness (STR) estimates the proximity of the cell’s pathway to a straight line with 100% corresponding to the optimal straightness in percentage and the mean angular displacement (MAD) which is the time average of absolute values of the instantaneous turning angle of the sperm head along its curvilinear trajectory in degree.^16^

**RNA extraction and cDNA synthesis of sperm cells. **Total RNA isolation was carried out on sperm cells according to the acid guanidinium thiocyanate-phenol-chloroform single-step extraction protocol as described earlier.^17^ Treatment of total RNA with RNAase-free DNAase (SinaClon BioScience Co., Karaj, Iran) was performed to avoid amplification of contaminating genomic DNA. The quality and integrity of the purified RNA was controlled by measurement of the A260/A280 nm ratio and by agarose gel electrophoresis. Only RNA samples showing integrity of the RNA by electrophoresis and exhibiting an A260/A280 ratio > 1.9 were used for synthesis of cDNA.

Total RNA was reverse transcribed into cDNA using M-MLV reverse transcriptase (SinaClon). The reverse transcription mixture was heated to 75 ˚C for 15 min to denature the RNA, and then stored at – 20 ˚C. 

**Real-time quantitative PCR analysis. **The levels of all three PPAR transcripts were determined by real time reverse transcriptase polymerase chain reaction (RRT-PCR). Glyceraldehyde-3-phosphate dehydrogenase (GAPDH) was selected as a housekeeping gene to normalize the difference of input load of cDNA between samples. Specific primers for PPARα, PPARγ, PPARβ/δ and GAPDH were designed using primer BLAST.^18^ The nucleotide sequences of the selected primer pairs and the length of amplified product are given in Table 1.

**Table 1:** Characteristics of used primers

**Gene**	**NIH GenBank accession No.**	**Product length (bp)**	**Primer sequence 5´- 3´**
**GAPDH**	NM_001190390.1	117	F:GTTCCACGGCACAGTCAAGG R:ACTCAGCACCAGCATCACCC
**PPARα**	XM_004007050.1	199	F:AGAACAAGGAAGCGGAAGTC R:ATCCCGTCTTTGTTCATCAC
**PPARγ**	NM_001100921.1	132	F:GAGGGCGATCTTGACGGGAA R:ACCTCTTTGCTGGGCTCCTG
**PPARβ/δ**	XM_004018768.1	153	F:CAACGAGGGGAGTCAGCACA R:AAGGGACTCCCAGCCGTTTG

GAPDH: Glyceraldehyde-3-phosphate dehydrogenase; PPAR: Peroxisome proliferator-activated receptors.

Real-time quantitative PCR (RT-qPCR) analysis was performed on Rotor-Gene Q 6000 System (Corbett Life Science, QIAGEN, Hilden, Germany) using SYBR premix EX Tag ІІ (Takara, Dalian, China). A volume of 1 µL cDNA was added to the Mix (0.5 µM of each specific primer, and 10 µL of SYBR premix EX Tag ІІReady Mix) in a total volume of 20 µL. An aliquot of each reaction mixture was subjected to electrophoresis in 1.5% agarose gel and stained with 0.5 μg mL^-1^ ethidium bromide. The relative quantification of three gene transcripts was determined in low and high motile sperm groups. Reaction condition was 95 ˚C for 5 min, 45 cycles of 95 ˚C for 40 sec, 63 ˚C for 30 sec and 72 ˚C for 30 sec. The PCR amplification was performed in triplicate for each sample with PPARs and GAPDH.

The cycle threshold (*CT*) values of the target genes (PPARα, PPARγ and PPARβ/δ) were normalized to those of the reference gene (GAPDH), and the relative quantification was performed according to Pfaffl method.^19^ Polymerase chain reaction efficiencies (*P*_eff_) were calculated according to a linear regression analysis with the LinReg PCR software (R^2^ value > 0.995)^20^ and the expression levels (E) of each gene were calculated according to the equation: 

*E= P*_eff _^(ΔCT)^

To ensure product homogeneity, the melting curve analysis was performed after the real time PCR procedure. The fluorescence signals were recorded continuously during temperature ramp (65 to 95 ˚C).

**Statistical analysis. **Differences between experimental group means were analyzed through one-way analysis of variance (ANOVA) with SPSS (Version 16; SPSS Inc., Chicago, USA) followed by Student’s *t*-test. All results are shown as mean ± SEM and differences were considered significant at *p* < 0.05. Pearson’s correlations were used to determine relationship between the level of gene expression and all sperm motion parameters for all three genes. R statistical environment (Version 2.15.2; R Development Core Team, Vienna, Austria) was used to estimate Pearson’s correlations and depict the figures.

## Results

**Sperm motility. **The results of CASA evaluation for sperm motility and sperm motility pattern are given in Tables 2 and 3. After separation on Percoll gradient, the remaining sperm phase in 45.00% Percoll, had significantly lower motile sperm and sperm cells with fast progressive motility (Table 2). The high motile sperm groups were also significantly better in sperm motility parameters such as VCL, VSL, VAP, LIN, WOB and STR than low motile sperm groups (Table 3). This result showed that the separation procedure was processed well.

**Table 2:** Mean ± SE of concentration, motility and progression of Percoll separated sperm samples (evaluated by CASA

**Groups ** **(n = 6)**	**Parameters**			**Progression (%)**			
	**Sperm density** **(10** ^6^ ** per mL)**	**Motile sperm ** **(%)**		**Fast progressive ** **(class A)**	**Slow progressive** **(class B)**	**Non-progressive** **(class C)**	**Non-motile ** **(class D)**
**High motile**	12.07 ± 2.56	76.40 ± 2.27*		58.53 ± 3.52†	12.01 ± 4.66	5.85 ± 0.51	23.60 ± 2.20*
**Low motile**	13.46 ± 1.73	58.49 ± 4.47		29.36 ± 2.41	16.34 ± 6.67	9.01 ± 3.85	44.00 ± 3.84

* indicate statistically differences in each column (*p* < 0.01)

† indicate statistically differences in each column (*p* < 0.0001).

**Fig. 1:**
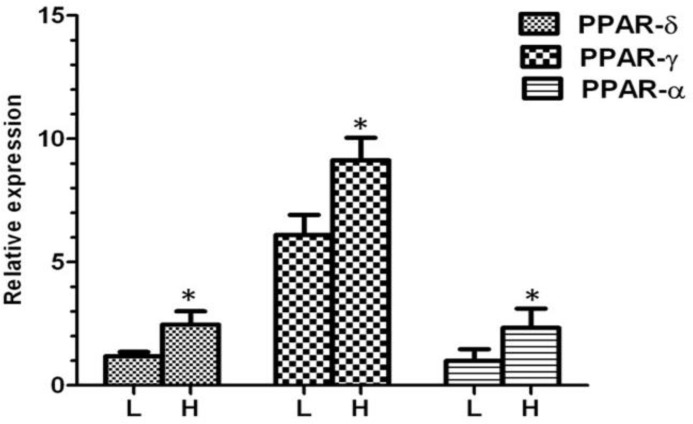
Relative expression of different genes in low and high motile sperm groups. L; low motile sperm, H; high motile sperm. Asterisk indicates significant difference between two groups

**Fig. 2:**
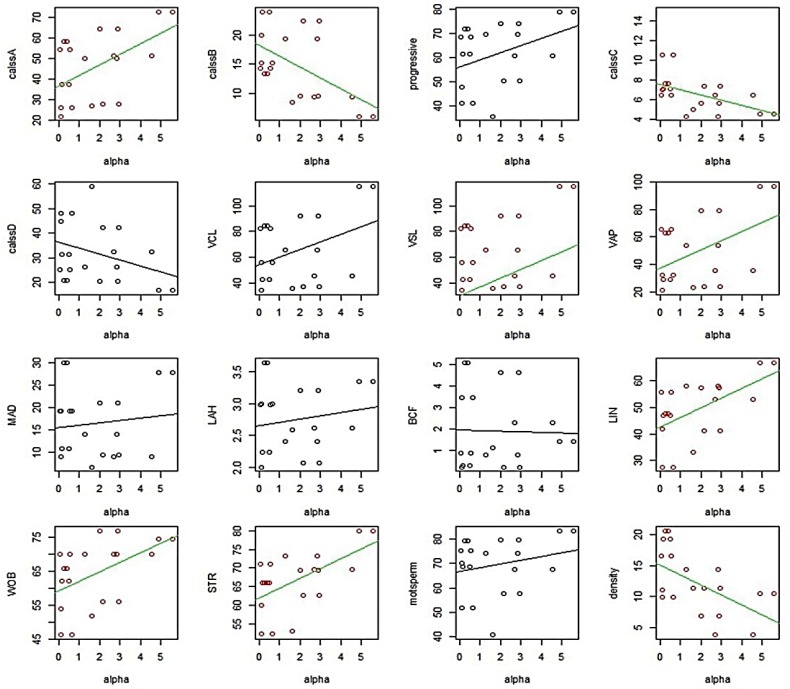
Graphs of regression analysis between PPARα mRNA abundance and sperm motility parameters. Motion parameters that had significant correlation with PPARα mRNA abundance are showed colored.

**Fig. 3.**
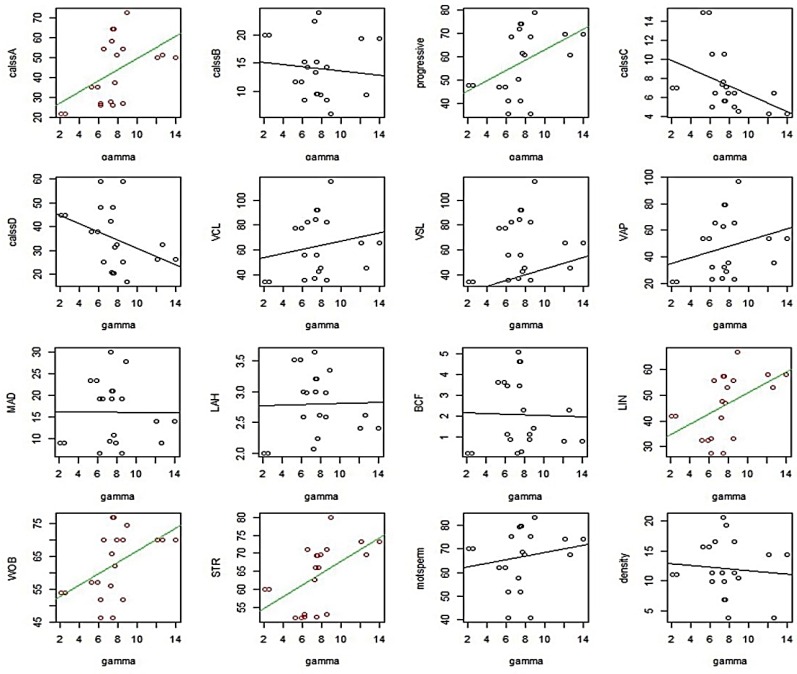
Graphs of regression analysis between PPARγ mRNA abundance and sperm motility parameters. Motion parameters that had significant correlation with PPARγ mRNA abundance are showed colored.

After separation, we analyzed the mRNA abundance of three genes between high and low motile sperm groups. As presented in Figure 1, the mean level of gene expression was significantly higher in high motile group than low motile, for PPARα, PPARγ and PPARβ/δ. In the next step and for more evaluation, the regression analysis was performed between the level of gene expression and all sperm motion parameters for all three genes. The results of this analysis showed that the mRNA abbundance for PPARα had a significant positive correlation with class A of sperm motility, VSL, VAP, LIN, WOB, STR and a significant negative correlation with class B and class C of sperm motility (Fig. 2). The level of mRNA for PPARγ was also showed a significant positive correlation with class A of sperm motility, percent of progressive motile sperms, LIN, WOB, and STR (Fig. 3). 

**Table 3:** Mean ± SE of sperm motility pattern parameters of Percoll separated sperm samples (evaluated by CASA

**Groups** **(n = 6)**	**VCL ** **(µm sec** ^-1^ **)**	**VSL ** **(µm sec** ^-1^ **)**	**VAP ** **(µm sec** ^-1^ **)**	**MAD ** **(°)**	**ALH ** **(µm)**	**BCF ** **(Hz)**	**LIN ** **(%)**	**WOB ** **(%)**	**STR ** **(%)**
**High motile**	80.93 ± 9.66*	57.13 ± 8.47†	65.58 ± 8.66*	20.20 ± 3.28	3.03 ± 0.18	2.49 ± 0.77	56.53 ± 2.56†	71.24 ± 1.59‡	71.52 ± 1.95†
**Low motile**	49.75 ± 7.78	23.65 ± 3.06	32.39 ± 5.66	13.86 ± 3.18	2.67 ± 0.26	1.73 ± 0.75	36.31 ± 3.48	54.64 ± 2.66	57.19 ± 2.97

VCL: Curvilinear velocity; VSL: Straight line velocity; VAP: Average path velocity; MAD: Mean angular displacement; ALH: Lateral head displacement; BCF: Beat cross frequency; LIN: Linearity; WOB: Wobble; STR: Straightness.

* indicate statistically differences in each column (*p* < 0.05);

† indicate statistically differences in each column (*p* < 0.01);

‡ indicate statistically differences in each column (*p* < 0.0001).

## Discussion

Mammalian spermatozoa are highly differentiated attractive cells because they have two different metabolic conditions in male (a quiescent metabolic state) and female (enhanced energy metabolism to accomplish complete functional maturation) genital tract and these cells are the only cells performing their function outside the male body. Sperm cells may attain access to their conspecific egg by mobilizing metabolic energy production in the form of ATP to drive motility. Sperm motility is essential for normal fertilization and one of the most important parameters in evaluating the fertilizing ability of ejaculated sperm. In this regard, correlations between the velocity of sperm move-ment or sperm motility and fertilization rates are proved.^21^

Ejaculated sperm retain a complex and specific, population of RNAs. It was recently proposed that these RNA transcripts may have important roles in sperm development, chromatin repackaging, and even zygote development.^22^ Studies on sperm RNA are available for humans,^23^ stallions,^24^ cattle^25^ and boars.^26^ The analysis of mRNA profiles in normal and abnormal sperm or ejaculate, is a growing field which can become a diagnostic and prognostic tool to evaluate male fertility and can lead to identify specific genetic pathways necessary for production of fertile sperm. For example, studies are currently underway to compare the genetic profiles of sperm samples from normal fertile men and teratozoospermic patients.^27^^,^^28^

In the present study, the mRNA abundances of all three PPAR isotypes were significantly higher in high motile sperm groups. The mRNA abundance of PPARγ was positively correlated to progressive motility. The PPARγ as a nuclear fatty acid receptor has an important role in the control of lipid and glucose or in general energy homeostasis.^29^ The PPARγ controls many different target genes involved in glucose homeostasis and lipid metabolism.^2^ Sperm energy metabolism is very complex and passes through the pentose phosphate cycle and catabolic pathways such as glycolysis and Krebs cycle. Studies by Aquila *et al*. have shown that insulin may be crucial in the management of sperm glucose metabolism since in autocrine fashion, it regulates G6PDH and glycogen synthase activities.^15^ It is showed that PPARγ activation regulates components of the phosphoinositide 3-kinase (PI3K) signaling cascade in various cell types.^30^ Aquila *et al*. examined the effects of a PPARγ-agonist rosiglitazone (BRL) treatment on PI3K-mediated signaling by evaluating the phosphorylation of the major downstream signal transducer, AKT^11^ since its phosphorylation has been correlated with its activity.^31^ Their results showed that increasing doses of BRL resulted in a significant increase in the AKT phosphorylation. BRL-stimulatory effect was also reduced by an irreversible PPARγ antagonist (GW9662). Therefore, they concluded that PPARγ -agonist stimulation of AKT was specifically mediated through PPARγ. The AKT plays multifunction roles in insulin action^31^ and sperm insulin activates PI3K pathway.^32^ Therefore, there is an interrelation between insulin and PPARγ. Insulin activated Glucose-6-phosphate dehydrogenase (G6PDH) in sperm and the activation is additive or synergistic to that of BRL. In these circumstances, G6PDH activity would theoretically increase glucose utilization because of improved insulin signaling in sperm as well as a cause of insulin sensitization. Therefore, it is speculated that PPARγ may be involved in the control of some sperm functions, perhaps by influencing the activity of PI3K. In agreement with our results, study of Santoro *et al.* showed that 15-deoxy-12, 14-prostaglandin J2 (PPARγ agonist) could increase sperm motility in pig.^33^ Their data showed that PPARγ was able to modulate the activity of G6PDH, the key rate-limiting enzyme in the pentose phosphate pathway (PPP**) **and the modulation was dose-dependent. The findings of De Amicis *et al.* proved that the effect of glucose on the fertilizing ability of spermatozoa appears to be mediated by its metabolism through the PPP.^12^ Considering all of these, it seems that the effect of PPARγ on sperm motility and viability is passing through energy metabolism.

In our study, the mRNA abundance of PPARα was also positively related to progressive motility. Although presence of PPARα in some spermatids (steps 7 and 8) and Sertoli cells has been approved in previous studies,^34^ as far as the authors of the present study concerned, this was the first report of PPARα presence in spermatozoa cells. PPARα regulates the beta-oxidation of lipids and may also regulate the fatty acid composition of phospholipids in germ cells.^35^ In a study by Douard *et al*., the lipid composition of spermatozoa was known to modulate mobility and viability of sperm cells.^10^ In their study, the modifications in lipid composition and lipid peroxidation were successively accompanied by decreasing in fertility, viability and sperm motility. These researchers stated that alterations in polyunsaturated fatty acids of the n-3, n-9, and n-6 series caused sperm membrane destabilization and led to changes in gamete viability, motility and fertilization capacity. Polyunsaturated fatty acids of the n-3 and n-6 series play a major role in the function and structure of gametes. Some studies in human spermatozoa have shown a positive correlation between motility and C22:6n-3 concentration.^36^ Dietary supplementation in these fatty acids of the n3 series, could improve the fertility of fresh fowl semen^37^ and the viability and morphology of pig spermatozoa.^38^ Using dietary supplementation in order to increase in the proportions of C22:4n-6 and C22:6n-3 in chicken spermatozoa, increased semen volume and number of spermatozoa per ejaculate.^39^ Therefore, considering the important role of PPARα in lipid metabolism and the special role of lipid composition and metabolism in sperm motility and viability, the effect of PPARα mRNA abundance on sperm motility may pass through this way. 

In conclusion, we showed for the first time that the PPARs mRNA is present in ram sperm cells and it was especially novel for PPARα and PPARβ, which were not reported earlier in sperm cells. The level of gene expression was correlated with some of the most important parameters of sperm motility pattern. These findings indicate that the products of PPARs gene expression can be involved in the physiology of sperm cell movement. More investigations will determine the role of these gene products for normal function of sperm cell. 
